# Editorial: Health and Disease in Free-Ranging and Captive Wildlife

**DOI:** 10.3389/fvets.2020.620685

**Published:** 2020-12-07

**Authors:** Robert J. Ossiboff, Francesco C. Origgi, Nicole I. Stacy

**Affiliations:** ^1^Department of Comparative, Diagnostic, and Population Medicine, College of Veterinary Medicine, University of Florida, Gainesville, FL, United States; ^2^Vetsuisse Faculty, Centre for Fish and Wildlife Health, University of Bern, Bern, Switzerland

**Keywords:** wildlife health, wildlife disease, wildlife, parasite, virus, clinical pathology, anatomic pathology

Wildlife populations of wide taxonomic breadth are declining globally at an astonishing rate (1–4). Habitat loss, environmental alteration and contamination, global movement of pathogens, excessive harvesting, and other predominantly anthropogenic factors have all been implicated as contributing to the global loss of biodiversity (1, 5–7). In light of these declines, the need for improved and increased knowledge about health and disease states of both free-ranging and captive wildlife species is greater than ever. This Research Topic highlights the current state of studies of health and disease in a wide range of animal species, including invertebrates, fish, amphibians, reptiles, avians, and aquatic and terrestrial mammals.

## Invertebrate Studies

Sea Star Wasting Syndrome (SSWS) is the cause of one of the largest marine wildlife die-offs ever recorded (8). Wahltinez et al. aimed to evaluate clinical pathology-related differences between sea stars affected by SSWS and clinically healthy individuals. Compared to clinically normal ochre sea stars (*Pisaster ochraceus*), SSWS affected sea stars had significant changes to coelomic fluid chemistry and higher coelomocyte counts. Bacteria were noted in 29% coelomic fluid samples from SSWS-affected sea stars but were absent in clinically normal sea stars. These chemistry and cytological findings in coelomic fluid of SSWS-affected sea stars provide insight into the pathophysiology of SSWS.

Giant African land snails (*Achatina fulica*) are one of the most popular pet snails globally. d'Ovidio et al. investigated the occurrence of parasites of giant African land snails kept as pets in southern Italy. Fecal examinations revealed the presence of multiple species of rhabditid nematodes, including *Rhabditella axei, Rhabditis terricola, Cruznema* sp., and *Pristionchus entomophagus*. The authors highlight that given the zoonotic potential of rhabditid parasites, this finding should be considered by those who maintain or treat this species in captivity.

## Fish Studies

Studies establishing baseline health parameters are an essential component of conservation studies (9). Malinowski et al. reported a comprehensive health assessment of the Atlantic goliath grouper (*Epinephelus itajara*). This study is the first to report a complement of health indices in free-ranging Atlantic goliath groupers from off the coasts of Florida, United States. The study also demonstrates that non-invasive blood samples can provide critical endpoint data and a wealth of health information that can be important for conservation studies.

Rubin et al. aimed to evaluate the role environmental parameters play in modulating the pathogenicity of *Tetracapsuloides bryosalmonae*, the causative parasitic agent of proliferative kidney disease (PKD). PKD is an emerging disease of salmonids, and has been implicated as a major factor contributing to noted decreases of wild brown trout (*Salmo trutta*) populations in Switzerland (10). The results of the investigation highlighted that water temperature ≥15°C was the parameter most significantly correlated with parasite prevalence and intensity. This finding further emphasizes the importance of temperature in salmonid PKD.

## Amphibian Studies

Chytrid fungi are important amphibian pathogens responsible for morbidity and mortality in free-ranging and captive frogs, salamanders, and caecilians (7, 11). Ossiboff et al. developed an automated dual-plex chromogenic RNAScope® *in situ* hybridization (ISH) assay for simultaneous detection and differentiation of *Batrachochytrium dendrobatidis* and *B. salamandrivorans*. The assay successfully identified organisms in tissue samples from different amphibian species preserved in formalin for up to 364 days and showed a remarkable sensitivity for the detection of *Batrachochytrium* spp. Furthermore, this assay highlighted the infection of dermal cutaneous glands in *B. salamandrivorans* infected caudates.

Hardman et al. investigated distal limb lesions of unknown etiology in free ranging Ozark hellbenders (*Cryptobranchus alleganiensis bishopi*) in Arkansas, United States. They found a high prevalence (93.2%) of hellbenders with digit lesions characterized by swelling and often ulceration. A single infectious etiology was not determined, though mass and *Batrachochytrium dendrobatidis* infection status were significantly and positively associated with the presence of the syndrome. A multifactorial etiology was presumed for this widespread and progressive disease syndrome.

In another study of hellbenders, Hopf et al. identified a novel species of *Exophiala* in a captive Eastern hellbender (*Cryptobranchus alleganiensis alleganiensis*). Although *Exophiala* are generally considered of opportunistic significance across taxa, they can be pathogenic in aquatic and semi-aquatic species. Given the progressive growth of lesions and chronic manifestation of this novel organism without overt evidence of underlying disease, the novel organism was suggested to be a primary pathogen in this Eastern hellbender.

## Reptile Studies

A study by Hoon-Hanks et al. investigated specific features of serpentovirus-associated disease in snakes, including the susceptibility of different types of captive snakes to serpentovirus infection and disease, the evaluation of viral genetic diversity involved in disease, and management practices associated with disease spread. Infection was more common in pythons and boas than in colubrids; associated respiratory signs were common in infected pythons, while rare to absent in other species. Detected serpentoviruses of different snake families showed wide genetic variability, suggesting that individual viruses might vary in their ability to infect divergent snake species.

Another respiratory infectious agent of snakes was reported by Walden et al.. The case report described *Raillietiella orientalis*, a pentastome parasite with a native range in Asia and Australia, in a free-ranging banded water snake (*Nerodia fasciata*) in Northern Florida. This parasite was first documented in invasive Burmese pythons in the Everglades and nine native species in Southern Florida 2 years prior in 2017, and another report further north in 2018 in native pygmy rattlesnakes (12, 13). The identification of this organism in Northern Florida documents an additional extension of the host range of this parasite, representing a potential emerging threat to native snake species in the southeastern United States.

Stacy et al. documented the role of *Caryospora*-like coccidial infections in morbidity and mortality in green sea turtles (*Chelonia mydas*). Partial genetic characterization of *Caryospora*-like coccidia collected from turtles in the southeastern US and one green turtle found in Hawaii was performed; sequences were compared to those of *Caryospora*-like coccidia from green turtles found in Australia. The authors reported that eight distinct genotypes were present in green turtles from the southeastern US, and phylogenetic analysis were suggestive of recent interoceanic dissemination of these parasites.

Mass mortalities of the Caribbean sharpnose puffer fish (*Canthigaster rostrata*: Tetraodontidae) were linked with episodic strandings of green sea turtles (*C. mydas*) exhibiting neurological signs or mortality in Costa Rica by González Barrientos et al.. Dietary-derived saxitoxins in whole fish, ingested fish, and brain, lung, kidney, and serum from affected turtles substantially exceeded human seafood safety thresholds. In contrast, endogenous tetrodotoxins were extremely low in fish and absent in turtle stomach contents. While juvenile green sea turtles are mainly herbivorous, they will opportunistically consume fish. Such fish consumption likely occurred during the described juvenile turtle strandings that coincided with puffer fish mass mortality events.

Weisbrod et al. reported on the diagnosis and management of a paratesticular cyst in a free-ranging green sea turtle (*C. mydas*). Their report is the first documentation of a reproductive-origin cyst in a reptile species which presented a unique clinical challenge given the uncommon diagnosis and the extent of the lesion. The report also describes medical management of acute anemia in this patient.

An exposure study by Harms et al. investigated the effects of Deepwater Horizon crude oil, dispersant, and oil-dispersant mixture on loggerhead sea turtle (*Caretta caretta*) hatchlings. Compared to the control group, exposed hatchlings had various clinicopathological derangements suggesting osmoregulatory, electrolyte, mineral, and hydration imbalances. The observed abnormalities document the acute pathophysiological mechanisms associated with oil and dispersant exposure in loggerhead sea turtle hatchlings. Furthermore, the results offer guidance for treatment modalities in oiled turtles and provide an understanding of impacts on a wildlife species during an oil spill response.

A number of members of the fungal order Onygenales have been characterized as significant pathogens in reptiles (14). Christman et al. reported a novel Nannizziopsiaceae within the order Onygenales associated with disease in a captive Galapagos tortoise (*Chelonoidis nigra*). The tortoise was presented for a clinical history of chronic progressive lethargy and inappetence. Imaging revealed the presence of a single large encapsulated pulmonary granuloma. Fungal culture, cytology, histopathology, and polymerase chain reaction were performed, and phylogenetic analysis provided solid evidence of a novel fungal species within the order Onygenales.

Wirth et al. investigated the occurrence of cutaneous skin lesions in resident populations of *Myuchelys latisternum* and *Emydura macquarii krefftii* in a natural pond in Australia. The prevalence of the lesions fluctuated with season from 0 to 77 and 68%, respectively. Blood biochemical parameters, body condition and activity levels were not significantly different between affected turtles and those without lesions. While investigations into an infectious cause were unsuccessful, the position of the lesions on the turtles suggested that the initiating factor was trauma associated with behavioral, seasonal, intra-species aggression.

A study by Palmer et al. compared the survival of adult three toed box turtles (*Terrapene mexicana triunguis*) at urban and rural sites in the state of Missouri, United States. While the reasons for most turtle deaths were unknown, the authors did find that odds of annual survival were 3.5 times greater for a turtle residing in the rural site as compared to the urban site. Their data suggest that even the largest urban parks may not be able to sustain healthy populations of adult box turtles, which has severe implications in the face of ongoing habitat loss and urbanization.

While multiple protocols for quantifying leukocytes in reptiles are available, critical comparisons of these protocols are lacking. Winter et al. compared two hematological methods, Avian Leukopet™ (LO) and total white blood cell estimates from eastern box turtle (*Terrapene carolina carolina*) blood films to evaluate agreement in total leukocyte counts between the two methods, as well as to document repeatability and reproducibility of the LO assay. The authors found significant differences between the two assays as well as variability in the LO assay, highlighting the need of a gold standard for reptilian leukocyte quantification.

## Avian Studies

Large scale oil spills can be a major source of morbidity and mortality in birds (15). Dannemiller et al. aimed to establish time-specific, descriptive blood analyte data following experimental sublethal oil exposure and subsequent rehabilitation in wild Ring-billed Gulls (*Larus delawarensis*). The authors reported that both sublethal oil exposure and aspects of captivity were associated with a mild non-regenerative anemia. The authors also reported that oiled gulls did not develop any clinicopathological derangements post-rehabilitation, consistent with a relatively low impact of current standard practices for rehabilitation in terms of morbidity in clinically stable and moderately oiled gulls.

Diao et al. reported on the concentrations of vitamins A and E in tissue samples from Anna's hummingbirds (*Calypte anna*). Nutritional diseases are the most common non-infectious disease in captive hummingbirds, and replicating a hummingbird's diet in captivity is challenging. Vitamin A and E levels were measured in different tissue types to provide baseline tissue concentrations which may be useful for conservation efforts to nutritional diseases of animals maintained or rehabilitated in captivity.

Finch trichomonosis is a disease of passerine birds caused by *Trichomonas gallinae*. This emerging disease has been associated with a 66% reduction of the British breeding greenfinch (*Chloris chloris*) population, while the breeding greenfinch population in the Netherlands has continued to grow. Rijks et al. investigated this discrepancy in population trends, and found that the frequency of fatal trichomonosis in the Dutch greenfinches did not differ significantly from that in Great Britain, with the parasite consistently being fatal in both bird populations. The results of the study concluded that trichomonosis is a threat concealed in Dutch breeding greenfinch census data.

Santos et al. investigated the prevalence of *Trichomonas* sp. in endangered Bonelli's eagle (*Aquila fasicata*) and several species of free-ranging and wild Colubriformes in areas of the Northwestern Iberian Peninsula via molecular testing of oro-esophageal swabs. Two genotypes appeared to be maintained in various species, with a high prevalence in Colubriformes. This study highlights the need to further investigate transmission dynamics and consider protective measures for Bonelli's eagles.

Another infectious agent of importance in avian species, *Sarcocystis falcatula*, was reported by Kirejczyk et al. in three penguins in managed care in the United States. Sarcocystosis resulted in fatal pneumonia in these intermediate aberrant hosts, as was confirmed by use of histology, immunohistochemistry, transmission electron microscopy, and PCR. Considerations for the route of infection included feces or sporozoite-containing fomite(s) shed by the definitive host, the Virginia opossum (*Didelphis virginiana)*, as well as contaminated water run-off or insects serving as mechanical vectors of opossum feces.

## Terrestrial Mammal Studies

Turchetto et al. carried out a broad and thorough comparative pathology investigation on sarcoptic mange, the most severe disease of wild Caprinae in Europe. The authors compared gross and histopathologic lesions of mange in different species, highlighting the known pathological and immunological aspects of the disease. The authors underlined the need to investigate the effect of immune responses on mange severity at an individual level, the main drivers in host–parasite interactions for different clinical outcomes, and the role of the immune response in determining the shift from epidemic to endemic cycle.

Blood lead levels (BLL) can be useful for monitoring environmental lead exposure in animals and investigating ecosystem health. Boesen et al. tested a specific BLL analyzer, Leadcare® Plus, against one of the gold standard measuring methods, inductively coupled plasma mass spectrometry in Scandanavian brown bears (*Ursus actos*). Their study shows that not only are Scandanavian brown bears highly exposed to environmental lead levels, but also that BLL analysis using Leadcare® Plus can be used for monitoring lead exposure if utilizing appropriate conversion equations.

Kehoe et al. described normal morphology of blood cells in four giant pandas (*Ailuropoda melanoleuca*), a vulnerable species and a charismatic member of zoological collections worldwide. Overall, similar leukocyte and platelet staining patterns to those reported in other mammals was observed. However, a unique mononuclear cell, with a moderately indented nucleus and shared cytochemical and ultrastructural characteristics of lymphocytes and monocytes was discovered. The combined cytochemical, immunocytochemical (CD3), and ultrastructural features of these unique cells most closely resemble those of monocytes, but the definitive cell lineage remains unknown at this time.

Babesiosis is a globally emerging arthropod-borne disease. Santoro et al. investigated the prevalence of *Babesia* spp. in free-ranging canids and mustelids in southern Italy, and found that the number of *Babesia* positive carnivores in wild carnivores in Italy is higher than previously presumed. Molecular characterization of the parasites identified both *B. vulpes* and badger-associated *Babesia* for the first time in Italy and the first time in a gray wolf (*Canis lupus*).

Ponthier et al. described a non-surgical option to modulate the testicular function of wild horses. A non-surgical castration of a captive wild Przewalski's (*Equus ferus przewalskii*) stallion was performed by anti-GnRH immunization. Immuno-neutering resulted in a decrease of total spermatozoa number and motility 1 month after primary vaccination. Six months post vaccination, serum testosterone concentrations had decreased; the total spermatozoa number was near zero with no motile spermatozoa, and no offspring were sired. On the basis of these results, the authors suggested that this technique could be considered as an alternative to surgical castration in wild horses.

Extrauterine pregnancies occur when the conceptus implants outside in a location other than the uterus. Hughes described a case of extrauterine abdominal pregnancy in a wild European rabbit, *Oryctolagus cuniculus*. While such pregnancies have been described in farmed and captive *O. cuniculus*, this is the first report of the condition in a wild individual. This condition has also rarely been described in other free-ranging lagomorphs, and should be considered as a possible differential diagnosis for abdominal masses in captive and free-ranging lagomorphs.

Neiffer et al. evaluated an immobilization protocol using etorphine, azaperone, and butophanol in free-ranging warthogs (*Phacochoerus africanus*). As the most commonly used drugs for immobilization in warthogs can be associated with falling and paddling during induction and recovery and require prolonged periods of time for recovery, there is a defined need for a rapid immobilization and recovery protocol. While the evaluated protocol was effective in immobilization of free-ranging warthogs with rapid induction and recovery, it was associated with significant cardio-respiratory changes that should be considered when used in potentially compromised warthogs.

A mandibular ossifying fibroma and multiple oral papillomas are reported in a roe deer (*Capreolus capreolus*) by Zürcher-Giovannini et al. Ossifying fibromas are benign fibro-osseous neoplasms that are rarely reported in veterinary medicine, and this report is the first documentation of this tumor in a cervid. Though species-specific papillomaviruses are frequently found in association with oral papillomas, no papillomaviral DNA or antigen were identified in association with the oral growths in this deer.

Gallina et al. reported on the presence of epitheliotropic viruses in free-ranging ruminants in Italy. Epitheliotropic viruses, including poxviruses and papillomaviruses, are known to infect wild ruminants globally. A collaborative network of hunters, wildlife rangers, and institutions was established to better understand interspecies transmission and clinical diseases caused by epitheliotropic viruses in Italy. Samples from chamois, red deer, and ibex were examined by ultrastructural, histological, and molecular methods. The results of the study demonstrated spread of parapoxvirus and papillomaviruses in Italy and the potential of these viruses to cause lesions in free-ranging ruminants.

## Aquatic Mammal Studies

*Otostrongylus circumlitis* (OC) infections are a significant cause of mortality in northern elephant seals (NES; *Mirounga angustirostris*) (16). Sheldon et al. investigated the diagnostic performance of complete blood count, serum chemistry, acute phase proteins, protein electrophoresis, and coagulation parameters for diagnosis of OC infections in NES. The diagnostic performance was highly accurate (area under the curve > 0.9) for albumin, albumin:globulin ratio, serum amyloid A, activated partial thromboplastin time, total bilirubin, and gamma-glutamyl transferase, consistent with useful indicators for systemic inflammation and DIC. These findings support previously reported clinical and gross pathological findings in NES infected with OC.

Goertz et al. reported comprehensive health assessment data for the 4 species of Alaskan ice seals, including bearded (*Erignathus barbatus*), ringed (*Pusa hispida*), spotted (*Phoca largha*), and ribbon (*Histriophoca fasciata*) seals. This publication will be a valuable reference for future investigations of mortality or disease events in these species, and for monitoring of spatial and temporal population dynamics in response to stressors such as environmental pollution and climate change.

In contrast to free-ranging pinnipeds, the common bottlenose dolphin (*Tursiops truncatus*) has been subject to population health monitoring for decades. In the review article by Barratclough et al., historical aspects, population threats, capture and handling techniques, recent technological and other methodological advancements, and the state of knowledge obtained by health assessment studies were discussed. The authors set the stage for future applications and emphasize the need for an integrative approach focused on interdisciplinary and interagency collaborations of such studies with bottlenose dolphins as a model species for marine mammal and ecosystem health and conservation.

One of the diagnostic techniques utilized in dolphin health assessments on an individual and population level is cardiac auscultation. Linnehan et al. developed an in-water cardiac auscultation technique and propose a method for its standardized use. Furthermore, the authors document highly prevalent, subtle heart murmurs of free-ranging and managed dolphins that have not been previously reported. The majority of detected murmurs were attributed to high velocity of blood flow similar to other athletic species.

A case of prolonged freshwater exposure in a bottlenose dolphin with characteristic skin lesions, corneal edema, and mild serum biochemical derangements was reported by Deming et al.. This individual was initially rescued from a freshwater lake, released to nearby brackish water, and found dead 12 weeks post-release due to fisheries interaction. Although the initially observed freshwater skin lesions resolved at time of necropsy, the corneal edema had worsened and presumably resulted in visual impairment; it remained undetermined whether this resulted directly from freshwater exposure or other trauma. This case report contributes to understanding freshwater exposure in dolphins and demonstrates complex associations of anthropogenic effects on their habitat.

An interesting case report by Câmara et al. described skeletal and cardiac rhabdomyolysis in a newborn Bryde's whale (*Balaenoptera edeni*). The observed rhabdomyolysis likely exacerbated pre-existing fetal distress upon stranding, resulting in death. While skeletal and myocardial damage has been documented in several cetacean species, this report is the first of its kind associated with a live-stranding in a newborn Bryde's whale.

Interactions with fisheries are a global threat and challenge to populations of aquatic animals, especially marine mammals (17). To determine the prevalence of fishery interactions with marine mammal death, Puig-Lozano et al. performed an 18-year retrospective study of stranded cetaceans in the Canary Islands; the authors document that 7.4% of cases with a known cause of dead were due to fishery interactions. The most frequent types of interactions were bycatch (i.e., ingestion of longline hooks, fishing net entrapments), chronic entanglements, and fishermen aggression. Such studies are important as a baseline for the development of informed conservation policies.

## Regional Wildlife Studies

Trimmel and Walzer reviewed the literature of infectious diseases in terrestrial wildlife species in Austria spanning 37 years. They found a continuous increase in publication frequency across the study period. Two-hundred twenty-six publications included 131 animal species, with the majority focused on parasitic infections, and lesser studies addressing viral, bacterial, and/or fungal infections. Interestingly, no publications on prions were found. Sixty-one percent of publications discussed publications with zoonotic potential. Although spatiotemporal associations could not be addressed in this study, this literature review provides an excellent summary of the state of wildlife research in Austria and identifies areas of future interest and need of attention.

## Conclusion

In light of recent and current declines in wildlife populations globally, there is a defined need for studies on states of health and disease in a wide taxonomic breadth of animals. Moreover, as we better understand the implications of host-pathogen interactions and the role of the ever-changing environment in shaping the disease ecology of free-ranging wildlife, there is a distinct demand for a renewed approach to characterizing mechanisms of disease and effects of stressors in non-domestic species. The papers published herein filled selective knowledge gaps while simultaneously highlighting the need for future, focused research in free-ranging and captive wildlife species: understanding basic physiology; identification and quantitative assessment of environmental factors and human stressors on wild populations; optimized preventive health measures, maintenance, and husbandry of non-domestic species in captivity; identification of novel infectious agents and their pathogenic and epidemiological significance in wild and captive populations; advancing medical and surgical management of certain conditions; mortality event investigations; and optimization of diagnostic procedures. While the editors of this Research Topic consider the pursuit a success given the variety and quality of papers and themes represented, we strongly encourage all researchers, veterinarians, and wildlife biologists to target studies that build upon these areas in need of attention for future research and to continue to publish their findings in the peer-reviewed and lay literature. Studies such as those contained in this Research Topic will be critical in the continued pursuit of global wildlife health and the preservation of biodiversity in the years to come as they can be essential tools for ongoing monitoring of populations, for identifying stressors and their effects on population dynamics, and ultimately for informing policy makers by building a basis for the development of conservation management practices that are based on high quality research answering specific population- or taxon-relevant questions.

## Author Contributions

RO, FO, and NS wrote and edited the article. All authors contributed to the article and approved the submitted version.

## Conflict of Interest

The authors declare that the research was conducted in the absence of any commercial or financial relationships that could be construed as a potential conflict of interest.

## References

[1] WWF Living Planet Report 2020 Bending the Curve of Biodiversity Loss. Almond REA, Grooten M, Petersen T, editors (2020).

[2] Butchart SHM Walpole M Collen B van Strien A Scharlemann JPW Almond REA. Global biodiversity: indicators of recent declines. Science. (2010) 328:1164–8. 10.1126/science.1187512 20430971

[3] Stuart SN Chanson JS Cox NA Young BE Rodrigues ASL Fischman DL. Status and trends of amphibian declines and extinctions worldwide. Science. (2004) 306:1783–6. 10.1126/science.1103538 15486254

[4] Böhm M Collen B Baillie JEM Bowles P Chanson J Cox N The conservation status of the world's reptiles. Biol Conserv. (2013) 157:372–85. 10.1016/j.biocon.2012.07.015

[5] Giam X. Global biodiversity loss from tropical deforestation. Proc Natl Acad Sci USA. (2017) 114:5775–7. 10.1073/pnas.1706264114 28550105 PMC5468656

[6] Roll U Feldman A Novosolov M Allison A Bauer AM Bernard R The global distribution of tetrapods reveals a need for targeted reptile conservation. Nat Ecol Evol. (2017) 1:1677–82. 10.1038/s41559-017-0332-2 28993667

[7] Scheele BC Pasmans F Skerratt LF Berger L Martel A Beukema W. Amphibian fungal panzootic causes catastrophic and ongoing loss of biodiversity. Science. (2019) 363:1459–63. 10.1126/science.aav0379 30923224

[8] Hewson I Sullivan B Jackson EW Xu Q Long H Lin C Perspective: something old, something new? Review of wasting and other mortality in Asteroidea (Echinodermata). Front Mar Sci. (2019) 6:406 10.3389/fmars.2019.00406

[9] Cooke SJ O'Connor CM Making conservation physiology relevant to policy makers and conservation practitioners. Conserv Lett. (2010) 3:159–66. 10.1111/j.1755-263X.2010.00109.x

[10] Wahli T Knuesel R Bernet D Segner H Pugovkin D Burkhardt-Holm P Proliferative kidney disease in Switzerland: current state of knowledge. J Fish Dis. (2002) 25:491–500. 10.1046/j.1365-2761.2002.00401.x

[11] Martel A Spitzen-van der Sluijs A Blooi M Bert W Ducatelle R Fisher MC *Batrachochytrium salamandrivorans* sp. nov. causes lethal chytridiomycosis in amphibians. Proc Natl Acad Sci. (2013) 110:15325–9. 10.1073/pnas.1307356110 24003137 PMC3780879

[12] Farrel TM Agugliara J Walden HDS Wellehan JFX Childress AL Lind CM Spillover of pentastome parasites from invasive burmese pythons (*Python bivittatus*) extends beyond the geographic range of pythons in Florida. Herp Rev. (2019) 50:73–6.

[13] Miller MA Kinsella JM Snow RW Hayes MM Falk BG Reed RN. Parasite spillover: indirect effects of invasive burmese pythons. Ecol Evol. (2018) 8:830–40. 10.1002/ece3.3557 29375757 PMC5773325

[14] Sigler L Hambleton S Paré JA. Molecular characterization of reptile pathogens currently known as members of the chrysosporium anamorph of nannizziopsis vriesii complex and relationship with some human-associated isolates. J Clin Microbiol. (2013) 51:3338–57. 10.1128/JCM.01465-13 23926168 PMC3811641

[15] Piatt JF Lensink CJ Butler W Nysewander DR Immediate impact of the “Exxon valdez” oil spill on marine birds. Auk. (1990) 107:387–97. 10.2307/4087623

[16] Colegrove KM Greig DJ Gulland FMD Causes of live strandings of northern elephant seals (*Mirounga angustirostris*) and pacific harbor seals (*Phoca vitulina*) along the Central California coast, 1992-2001. Aquat Mamm. (2005) 31:1–10. 10.1578/AM.31.1.2005.1

[17] Reeves RR McClellan K Werner TB Marine mammal bycatch in gillnet and other entangling net fisheries, 1990 to 2011. Endanger Species Res. (2013) 20:71–97. 10.3354/esr00481

